# Effectiveness of Web-Based Cognitive Behavioral Therapy for Depression: A Systematic Review of Randomized Controlled Trials

**DOI:** 10.7759/cureus.73905

**Published:** 2024-11-18

**Authors:** Yousef A Alzilfi, Rawan A AlMalki, Abdullah H AlMuntashiri, Jameela A AlMathami

**Affiliations:** 1 Family Medicine, Prince Mansour Military Hospital, Taif, SAU; 2 Family Medicine, Al-Rawda Health Center, Taif, SAU

**Keywords:** cognitive behavioral therapy, depression, interventions, randomized controlled trials, web-based

## Abstract

Evidence-based practices were more effective in managing mental health disorders when compared to traditional, non-evidence-based approaches. Web-based cognitive behavioral therapy (CBT) interventions offer a wide variety of advantages among depressed patients as they offer a sense of anonymity, privacy, and accessibility. This systematic review of randomized controlled trials (RCTs) aims to estimate the efficacy of several types of web-based interventions among patients with depression symptoms. This review was conducted according to the principles of the Cochrane Handbook for Systematic Reviews of Interventions and reported following the Preferred Reporting Items for Systematic Reviews and Meta-Analyses (PRISMA) guidelines. We searched the literature using five databases: PubMed, Medline, Ovid, Cochrane CENTRAL, and Scopus. Key terms for depression and web-based CBT included "Depression", "Cognitive behavioral therapy", "CBT", "Effectiveness", "Internet", and "Web-based". The included studies were published between 2019 and 2024 and assessed the risk of bias (ROB) using the ROB2 tool for RCTs. This search strategy yielded 2902 records, of which 1946 were duplicates and removed. The remaining 956 records underwent screening of their titles and abstracts, and 900 records were excluded. The full texts of the remaining 56 records were retrieved; 45 of them were excluded since 13 had the wrong study design, 10 had inappropriate outcomes, 12 had inappropriate populations, and 10 were out of the date range. All the included studies were 11 RCTs. Most studies assessed the efficacy of internet-based CBT (ICBT) or web-delivered CBT (wCBT). Control groups included the waitlist control (WLC) group and the treatment as usual (TAU) group, and one study included an active control group. Six studies compared the effects of guided or self-guided ICBT with control, three compared wCBT with control, one used internet self-guided intervention, and one employed an internet-based Functional Depression (iFD) program. Interventions were generally self-guided or guided by laypersons, nonspecialists, or automated systems. It can be concluded that despite the significance of most interventions favoring internet-based interventions compared to control groups, many limitations existed in the included studies, making it unclear whether the interventions would prove conclusive and reliable benefits. Therefore, more studies should be conducted on larger sample populations with prolonged duration.

## Introduction and background

Depression is one of the most widespread mental health disorders globally, impacting approximately 19.7% of individuals over the age of 16 [1]. The prevalence of depression elevated significantly during the COVID-19 pandemic, with rates more than tripling compared to pre-pandemic levels [2]. Validated questionnaires and structured interviews diagnose depression, such as the Patient Health Questionnaire-9 (PHQ-9) [3] and the Hamilton Depression Rating Scale (HDRS) [4]. Self-report instruments are commonly used in studies, although these may not always detect the full extent of the condition [5]. Recently, many programs have been developed to treat depression. For instance, physical activity interventions have shown medium to large effects on depression symptoms [6]. In addition, various psychological treatments, including cognitive behavioral therapy (CBT) and interpersonal therapy, have emerged as effective approaches for depression management [7].

CBT has been considered an effective treatment intervention for major depressive disorder (MDD). For instance, one study reported that 75.9% of participants achieved remission following the CBT intervention. This shows that CBT can result in high rates of depression remission [8]. Over the past decades, providing effective and affordable care to individuals suffering from various mental health problems, including depression, is essential. The internet has opened new avenues for mental health professionals [9]. It was found that web-based interventions are increasingly employed to support patients, as studies showed significant positive effects on outcomes, specifically, efficacy compared to usual care or no care [10].

Web-based CBT interventions offer a wide variety of advantages among depressed patients. First, accessibility is highly important, especially for participants with limited access to in-person care [11]. In addition, the online format of web-based CBT may offer a sense of anonymity and privacy, which could help participants who are hesitant to seek in-person mental health services to be involved in treatment. Moreover, these interventions could be easily disseminated and scaled to reach a larger population than traditional in-person therapy [12].

Evidence-based practices were more effective in managing mental health disorders when compared to traditional, non-evidence-based approaches [13]. For instance, a previous systematic review and meta-analysis revealed that web-based CBT interventions had a moderate to large influence on improving symptoms of grief and post-traumatic stress disorder among individuals, with stable effects over time [14]. However, findings regarding the efficacy of these interventions among patients were not well-established in the literature, suggesting a more rigid program aligned with patient's needs.

This systematic review of randomized controlled trials (RCTs) aims to estimate the efficacy of several types of web-based CBT interventions among patients with depression symptoms. The findings of this systematic review will help guide practitioners and researchers on the advantages and disadvantages of these interventions based on the response and findings of each included study.

## Review

Methodology

This systematic review aims to evaluate the efficacy of internet-based interventions in attenuating depression symptoms among the adult population. The systematic review included subjects who are aged >18 having major depressive disorder with mild or moderate depression episodes. Several types of internet-based interventions were employed in the included studies.

The study was conducted according to the principles of the Cochrane Handbook for Systematic Reviews of Interventions and reported following the Preferred Reporting Items for Systematic Reviews and Meta-Analyses (PRISMA) guidelines [15].

Search Strategy

We searched the literature using five databases: PubMed, Medline, Ovid, Cochrane CENTRAL, and Scopus. Key terms for depression and web-based CBT included "Depression", "Cognitive behavioral therapy", "CBT", "Effectiveness", "Internet", and "Web-based" and their synonyms. We targeted studies that measured the effectiveness of the intervention by reporting attenuating depressive symptoms and using validated depression assessment tools. We focused on studies designed as RCTs. The combined search string was as follows: ("Cognitive behavioral therapy" [Title/Abstract]) OR (CBT [Title/Abstract])) AND (Depression[Title/Abstract]) AND ((Internet[Title/Abstract]) OR Web[Title/Abstract])).

Eligibility Criteria

The included studies were published between 2019 and 2024 and aimed to assess the effectiveness of internet- or web-based CBT interventions among patients with major depressive disorder. Only RCTs with two arms were included. Only studies that included quantifiable measures for depression by a validated assessment tool were selected. The population groups included males and females. Studies published in English were retrieved for this review.

Exclusion Criteria

The excluded studies were as follows: (1) studies with no full-access link; (2) studies with inappropriate objective/outcome; (3) studies that include web-based interventions blended with face-to-face treatment; (4) studies that have interventions delivered only through smartphones; (5) studies that were conducted among patients with subthreshold depression and physical illness, such as cancer or chronic medical conditions; (6) study types such as case reports, letters, observational studies, review articles, and systematic review articles; and (7) duplicate studies found in multiple databases or sources.

Data Extraction

The following data were collected from each RCT: authors' names, years, country, population characteristics, types of web-based CBT interventions, and the efficacy of the intervention measured by decreasing depressive symptoms, as well as PHQ-9 scores or any other validated depression-related score. The PHQ is a self-administered version of the Primary Care Evaluation of Mental Disorders diagnostic instrument for common mental disorders. The PHQ-9 scores each of the nine diagnostic criteria for major depression in the Diagnostic and Statistical Manual Fourth Edition as "0" (not at all) to "3" (nearly every day) [16]. The total PHQ-9 scores of 5, 10, 15, and 20 represent cutpoints for mild, moderate, moderately severe, and severe depression, respectively [17].

Assessment of the Risk of Bias (ROB) in the Included Studies

We assessed the ROB using the ROB2 tool for RCTs [18], as all included studies were found to be RCTs. The ROB2 tool comprises five domains: randomization, deviations from the assigned treatment, missing data, measurement of the outcome, and selective reporting of the outcomes and results. Moreover, the overall ROB is assessed by selecting the highest level of ROB out of the five domains. The data was visualized using the robvis tool [19].

Results

Study Selection

**Figure 1:**
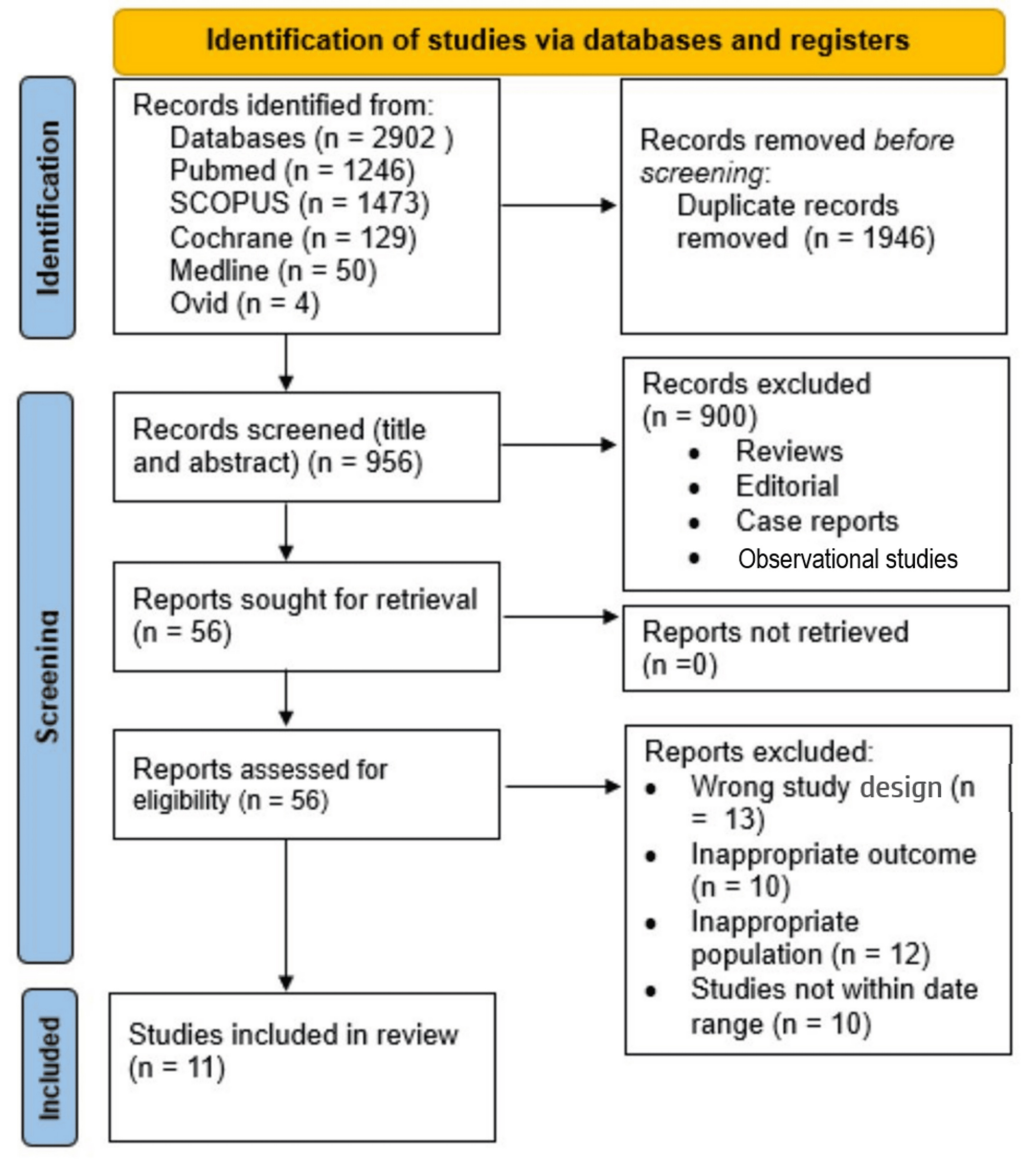
PRISMA flowchart of the selection process PRISMA: Preferred Reporting Items for Systematic Reviews and Meta-Analyses

The search strategy yielded 2902 records, of which 1946 were duplicates and removed. The remaining 956 records underwent screening of their titles and abstracts, and 900 records were excluded. The full texts of the remaining 56 records were retrieved and assessed for eligibility for inclusion in the present systematic review. Out of them, 45 were excluded as follows: 13 had the wrong study design, 10 had inappropriate outcomes, 12 had inappropriate populations, and 10 were not within the date range (2019-2024). Finally, 11 RCTs met our inclusion criteria and were selected for inclusion in our systematic review (Figure 1) [20-30].

The Basic Characteristics of the Included Studies

All the included studies were RCTs. Three studies were conducted in the USA [20,23,30], two studies were conducted in Sweden [25,29], and one study each was conducted in Germany [28], the UK [26], Brazil [22], Canada [27], China [21], and the Netherlands [24]. The studies recruited participants from communities (two studies), psychiatric clinics or hospitals (four studies), and university settings (four studies), and one study recruited a specific population: an Arabic-speaking immigrant population.

Most studies assessed the efficacy of internet-based CBT (ICBT) or web-delivered CBT (wCBT). Control groups included the waitlist control (WLC) group (n=7) and the treatment as usual (TAU) group (n=3), and one study included an active control group. Six studies compared the effects of guided or self-guided ICBT with control, three compared wCBT with control, one used internet self-guided intervention, and one employed an internet-based Functional Depression (iFD) program. Interventions were generally self-guided or guided by laypersons, nonspecialists, or automated systems. In guided ICBT groups, guidance was provided by trained lay coaches [20], nonspecialists [21], counselors [27], and therapists [29], while other programs were self-guided or fully automated. The proportion of participants taking antidepressants varied across studies, ranging from 2.1% to 83% [20-30]. Although almost all studies relied on the PHQ-9, only one study used the Hospital Anxiety and Depression Scale (HADS) to evaluate treatment efficacy [26].

The various interventions included in the studies showed variable internet-based CBT structures designed to improve depressive symptoms. For instance, "Empower@Home" includes nine sequenced sessions that blend videos, text, exercises, and home practices; ICBT intervention consists of seven modules focused on cognitive restructuring and problem-solving skills, with follow-up from six weeks to 12 months; Deprexis includes 10 primary content modules and a review module; Thrive ICBT is an automated program that provides three video-based modules with 10 lessons on CBT techniques over an eight-week follow-up period; ICare Prevent is a transdiagnostic intervention tailored to individual needs; Feel Stress-Free includes four automated behavioral relaxation activities incorporating mood tracking and thought challenging and a relaxing minigame over six weeks; CBT (MVC) intervention combines 12 mental health modules; and the iFightDepression (iFD) tool is a self-guided program consisting of six core workshops comprising written information, worksheets, exercises, and a mood rating (Table 1) [20-30].

**Table 1:** Characteristics of the included studies wCBT: web-delivered cognitive behavioral therapy; MDD: major depressive disorder; ICBT: internet-based cognitive behavioral therapy; WLC: waitlist control; DSM-V: Diagnostic and Statistical Manual of Mental Disorders, Fifth Edition; PHQ-9: Patient Health Questionnaire-9; MINI: Mini-International Neuropsychiatric Interview; TAU: treatment as usual; HADS: Hospital Anxiety and Depression Scale; VU: Vrije Universiteit; UvA: Universiteit van Amsterdam

Study	Country	Aim	Study design	Population	Exclusion in the studies	Intervention	Type of intervention	Comparison	Number of patients who took antidepressants (%)
Xiang et al., 2024 [20]	USA	To evaluate the efficacy of a novel wCBT program, "Empower@Home," supported by trained lay coaches against a waitlist attention control	Parallel RCT	Sample size (N)=70. Population source: participants were recruited from research volunteer registries, social media advertisements, and referrals from community agencies. Inclusion criteria: (1) ability to read and speak English, (2) residency in Michigan, and (3) being aged at least 60 years and having elevated depressive symptoms at screening	(1) Participants with dementia, a psychotic disorder, moderate to high risk of suicide, or a terminal illness; (2) a current substance use disorder or uncorrected severe vision impairment (e.g., blindness); or (3) receiving or planning to receive psychotherapy during the trial. Participants without a computer or internet access were provided with a cellular tablet at no cost during the trial	wCBT Empower@Home contains nine sequenced sessions, drawing from the CBT manual for working with older people. Users accessed self-help therapeutic tools and lessons through a dedicated website. Once logged onto the website, they navigated the web-delivered sessions through a blend of brief videos, narrated text pages, short exercises, and offline home practices. Each session was designed to be completed independently in approximately 20-30 minutes. Users completed sessions in their homes and could complete sessions during their weekly coaching calls based on their preferences. The participants were encouraged to complete one session weekly	The layperson supported the program	WLC	Treatment=11 (31%). Control=14 (40%)
Lin et al., 2023 [21]	China	To evaluate the efficacy of ICBT for depressive symptoms in patients with MDD	Two-arm and nonblinded RCT	Sample size (N)=84. ICBT=40. WLC=44. Population source: the Department of Depressive Disorder of Shenzhen Kangning Hospital and the Department of Psychiatry of Shenzhen Nanshan Center for Chronic Disease Control. Inclusion criteria: (1) participants aged 18-60 years; (2) meeting the criteria for MDD in the DSM-V; (3) a score of more than 5 on the PHQ-9, indicating the presence of at least mild depressive symptoms; (4) if taking an antidepressant being on a stable dose for at least four weeks; and (5) access to an internet-connected smartphone	(1) History of neurological diseases; (2) moderate or high risk of suicide according to section C of the MINI; (3) currently undergoing physiotherapy or psychotherapy treatment;(4) pregnancy or breastfeeding; (5) severe physical disease; (6) psychosis or bipolar disorder; and (7) substance use disorder	ICBT. The program consists of seven treatment modules corresponding to conventional face-to-face CBT courses of treatment that aim to teach users how to manage their depressive symptoms using CBT skills, including cognitive restructuring, problem-solving, and relapse prevention	ICBT support by nonspecialists	WLC	ICBT=22 (55%). WLC=26 (59%)
Lopes et al., 2023 [22]	Brazil	To assess whether Deprexis is effective in reducing depressive symptoms and the general psychological state of Brazilian users with moderate and severe depression in comparison with a control group	Parallel RCT	Sample size (N)=189. Mean age=36.44. SD: 11.52 years old; range=19-71. Male=31 (16.4%). Female=158 (83.6%). Population source: a website, a fan page on Facebook, an Instagram account, and a Twitter profile were set up to provide basic information about the research procedures, about the internet-based intervention, and to recruit participants. Eligible criteria: (1) participants at least 18 years of age; (2) participants with regular access to the internet; (3) residents of Brazil; (4) those who presented clinically relevant depressive symptoms defined as a score of at least 10 on PHQ-9; and (5) participants diagnosed with a depressive disorder or dysthymia	(1) Candidates who presented other severe psychiatric symptoms that should be the primary focus of clinical attention (i.e., severe psychotic symptoms, manic episodes, severe substance abuse, and severe obsessive-compulsive disorder); (2) showed the potential to harm themselves or others; (3) had severe suicidal ideation; and (4) were psychiatric patients in the process of adaptation to medication	Deprexis is designed to help people cope with and overcome depressive symptoms. It is based on the principles of CBT and consists of 10 primary content modules plus one brief review module. Intervention: TAU plus immediate access to Deprexis for 90 days=94	Internet-based, self-guided intervention	TAU and delayed access to Deprexis after eight weeks (n=95)	Pharmacology intervention: 20 (21%). Control: 26 (27%). Psychotherapy intervention: 8 (9%). Control: 7 (7%)
Stuart et al., 2022 [23]	USA	To compare the effectiveness of ICBT for depression to that of TAU alone	Randomized efficacy trial	Sample size (N)=302. Age=47.2±16.1, 81.1% female patients, and 51.7% non-Hispanic White patients. Population source: Southern California Kaiser Permanente, San Bernardino County Area, Department of Family Medicine. Eligibility criteria: (1) participants ≥18 years old; (2) English-speaking; and (3) having an in-person visit with their primary care provider within the past two weeks and current depression. Alternatively, a related diagnosis is associated with that visit	Patients with a current diagnosis of (1) schizophrenia (or related disorder); (2) delirium; (3) bipolar disorder or any neurocognitive disorder; (4) intellectual disability or personality disorder; or (5) active substance use disorder	Thrive ICBT was designed to teach patients CBT techniques primarily through videos. It is a fully automated ICBT program with three video-based modules containing 10 lessons using behavioral, cognitive restructuring, and social skills training. Participants were randomized to receive either TAU or additional access to Thriveactivation. Treatment (N)=142	Fully automated ICBT program	TAU, control (n=154)	Intervention (ICBT+TAU): 71.6% (106). Control (TAU): 76% (117)
Karyotaki et al., 2022 [24]	Netherlands	To investigate the effectiveness of ICare Prevent against TAU in reducing mild to moderate depression and/or anxiety symptoms among college students	Two-arm randomized controlled superiority trial	Sample size (N)=100. Population source: Dutch universities, namely, the VU and UvA. Eligibility criteria: (1) 18 years of age or older; (2) enrolment as a bachelor's or master's student in a Dutch college; (3) fluency in Dutch or English; (4) mild to moderate symptoms of depression defined by scoring above the cutoff score of 4 on the PHQ-9; and (5) provision of written informed consent before participation	(1) Diagnosis of bipolar disorder according to the MINI; (2) moderately severe/severe depressive symptoms as defined by scoring above the cutoff score of 14 on the PHQ-9; (3) receiving psychological treatment for depression or anxiety in the past 12 months, and/or (4) no internet connection	ICBT. It is a transdiagnostic individually tailored intervention called "ICare Prevent," a new program based on established ICBT components (e.g., problem-solving, behavioral activation, and cognitive restructuring). Participants were randomized at an individual level (1:1 ratio). Furthermore, they were stratified by recruitment location (VU and UvA). ICBT (n=48)	Guided ICBT	TAU (n=52)	Six months follow-up (ICBT)=1 (2.1%); 12 months follow-up=2 (4.2%)
Lindegaard et al., 2021 [25]	Sweden	To examine the efficacy of ICBT in an Arabic-speaking immigrant population	Randomized controlled pilot trial	Sample size (N)=59. Age (years): M (SD)=37.5 (11.4). Range: min-max=20-69. Gender: n (% male)=34 (58%). Population source: Arabic-speaking immigrant population living in Sweden. Eligibility criteria: (1) ability to read and write Arabic fluently; (2) having elevated symptoms of depression and anxiety as determined by their scores on the PHQ-9 and GAD-7, respectively; (3) currently residing in Sweden; and (4) above 18 years	(1) Having a severe mental illness such as bipolar disorder or schizophrenia; (2) suicidal ideation; (3) substance or alcohol abuse; and (4) ongoing psychological treatment	ICBT (N=30) participants were randomly allocated to either an eight-week ICBT treatment or a WLC condition in a 1:1 ratio	Guided ICBT	WLC (n=29)	Prior psychological treatment: n (% yes)=9 (15%)
McCloud et al., 2020 [26]	UK	To evaluate for the first time the effectiveness of a self-guided mobile app, Feel Stress-Free, for the treatment of depression and anxiety symptoms in students	A web-based randomized unblended controlled trial	Sample size (N)=168. The mean age of all 168 participants was 24.3 years (SD: 6.71; range: 18-54 years). 82.7% (139/168) of the participants were female and 61.9% (104/168) were undergraduate students. Population source: a total of four universities partnered with Thrive Therapeutic Software Limited agreed to take part: University College London (UCL), School of Oriental and African Studies University of London, University of Buckingham, and University of Roehampton. Eligibility criteria: (1) eligible participants were aged 18 years or over and scored 8 or above on one or both subscales of the HADS, indicating at least a possible case of depression and anxiety; (2) were currently a student at one of the four partnered universities; and (3) had access to an Apple or Android phone or tablet or a computer with Firefox, Safari, or Chrome	NA	CBT-based mobile app Feel Stress-Free. The app consists of CBT-based activities to help users manage symptoms of depression and anxiety. The app comprises four behavioral relaxation activities: calm breathing, mindfulness-style meditation, deep muscle relaxation, and self-hypnosis; one cognitive activity incorporating both mood tracking and thought challenging; a relaxing minigame; and a feature for positive messages in a bottle. Compared with a WLC, university students self-identified as experiencing symptoms of anxiety or depression and were randomized to six weeks of intervention (n=84) or control (n=84), unblinded	Self-guided application	WLC (n=84)	NA
El Morr et al., 2020 [27]	Canada	To assess the effectiveness of an eight-week web-based mindfulness and CBT program in reducing symptoms of depression, anxiety, and stress (primary outcomes) and increasing mindfulness (secondary outcome) within an RCT with undergraduate students at a large Canadian university	RCT	Sample size (N)=160. Male=32. Female=125. Mean age of 22.55 years. Population source: a large Canadian university. Eligibility criteria: (1) undergraduate students who were at least 18 years of age; (2) reported English language fluency; (3) self-rated high confidence in completing the study; and (4) were actively enrolled in an undergraduate program. Using a computer and smartphone and internet literacy were considered de facto skills	(1) Substance abuse or episodes of psychotic behaviors during the month prior to the trial	MVC intervention was eight weeks in duration. The intervention comprised three components: (1) 12 student-specific mental health modules conveyed by online video; (2) three anonymous discussion boards dedicated to depression, anxiety, and stress; and (3) an anonymous 20-minute group-based live videoconference led by a moderator (a counselor with a master's degree in psychology and training in mindfulness) during which students could raise and discuss topics covered in the modules MVC (n=80). Participants were randomly allocated to a web-based guided mindfulness-CBT condition (n=80) or to a WLC condition (n=80). Participants in all groups completed online questionnaires at baseline (T1) and eight weeks (T2)	Web-based guided	WLC (n=80)	NA
Oehler et al., 2020 [28]	Germany	To assess the acute and long-term antidepressant efficacy of a six-week, guided, web-based self-management intervention building on the principles of CBT (iFightDepression tool) for patients with depression compared with web-based progressive muscle relaxation as an active control condition	RCT	Sample size (N)=348 patients with mild to moderate depressive symptoms. Age M (SD): intervention: 42.9 (12.4); control: 41.7 (12.4). Female n (%): intervention: 137 (79.2%); control: 136 (78.3%). Population source: participants were recruited throughout Germany via the website, social media channels, appearances in other media, and newsletters of the German Depression Foundation (DF). Eligibility criteria: (1) outpatient status, a diagnosis of depressive disorder with presently mild or moderate severity (F32.0, F32.1, F33.0, and F33.1) or dysthymia (F34.1) according to the MINI and PHQ-9 (score 5-14, indicating mild-to-moderate symptoms); (2) aged 18 years and above; (3) sufficient language skills to meet the study requirements, as well as internet access; and (4) outpatient status was included so patients could be referred to their local care provider in a crisis	(1) Dementia; (2) drug or alcohol abuse within the last six months; (3) drug or alcohol addiction, schizophrenia, manic episodes, bipolar disorder, or obsessive-compulsive disorder (all according to the MINI); (4) known personality disorders (F60.2 and F60.31); (5) acute suicidal tendencies; (6) severe somatic disorders requiring immediate treatment; and (7) pregnancy and participation in another clinical trial within the past four weeks	iFD. The iFD tool is a guided web-based self-management tool based on CBT principles. It includes six core workshops, each comprising written information, worksheets, exercises, and a mood rating. Intervention group (n=173). Participants were recruited online and randomly assigned to one of the two intervention arms	Web-based guided	Active control condition (PMR) control group (n=174)	Intervention: 115 (66.5%). Control: 108 (62.1%)
Johansson et al., 2019 [29]	Sweden	To examine the symptom levels of the participants six and 12 months after ending ICBT treatment	RCT	Sample size (N)=54. Mean age (years)=39. Population source: patients at psychiatric clinics in Sweden are normally referred from primary care in cases where the psychiatric treatment is expected to be long and when more special-led care is required. Eligibility criteria: (1) being a minimum of 18 years of age; (2) a primary diagnosis of MDD, either a single episode or recurrent episodes (in case of comorbidity, if using antidepressants, a minimum of 30 days of stable dosage prior to inclusion was required); (3) participants should agree to refrain from psychotherapy during the participation in the study; (4) be fluent in Swedish; and (5) have access to a computer, smartphone, or tablet with internet connection	(1) An ongoing alcohol or substance abuse disorder;(2) being assessed as a high-risk suicidal patient; (3) being actively engaging in self-harm; and (4) having a current eating disorder, bipolar disorder, or ongoing psychotic symptoms	ICBT N=27. Participants were randomized into two groups: (1) an experimental group that received therapist-supported internet-delivered CBT for depression and (2) a WLC group	Guided ICBT	WLC (n=27)	45 (83%)
Schure et al., 2019 [30]	USA	To evaluate the effectiveness of an ICBT intervention called Thrive, which was designed to enhance engagement when delivered as a fully automated, stand-alone intervention to a rural community population of adults with depression symptoms	RCT	Sample size (N)=343. Female (290/343, 85%) and Caucasian (319/343, 93%), with a mean age of 42.9 (SD: 13.3) years. Population source: rural community population of adults with depression symptoms. Inclusion criteria: (1) adults aged ≥18 years with mild to severe depression severity (PHQ-9 score >5); (2) Montana residency; (3) a valid email address; and (4) regular access to broadband internet via a computer, tablet, or smartphone. At enrollment, potential participants who indicated recent suicidal ideation (PHQ-9 item 9 score >0) were asked to confirm that they could stay safe, and those responding that they could not be considered ineligible	(1) Three individuals provided invalid email addresses; (2) participation and data from two control participants were discarded, as they were accidentally provided access to the intervention immediately; (3) missing baseline data on the covariate; and (4) currently receiving psychosocial therapy for depression	ICBT RCT compared the efficacy of the fully automated, stand-alone Thrive intervention to a WLC in reducing depression symptoms among adults. Participants were recruited from communities across Montana and immediately randomized 1:1 to the intervention group (Thrive) or a WLC group (delayed access to Thrive until the eight-week follow-up assessment) after meeting the inclusion criteria and providing electronic informed consent on the study website (n=181)	Self-guided ICBT	WLC (n=162)	Total=202 (58.9%). Intervention: 103 (56.9%). Control: 99 (61.1%)

Table 2 reports the outcomes of the included studies regarding the efficacy of web-based interventions for depression, focusing on the changes in PHQ-9 scores pre- and post-intervention for the total population recruited, treatment, and control groups. Most interventions demonstrated significant reductions in depressive symptoms compared to control groups. This was evident in the following: ICBT (p<0.001, p=0.002) [21,23], Thrive ICBT (p<0.001) [30], Feel Stress-Free app over six and eight weeks (p=0.04, 0.006) [26], mindfulness CBT video-assisted program (p<0.001) [27], and iFD tool (p=0.01) [28]. The studies indicated that various CBT internet interventions were generally effective in reducing depressive symptoms, with significant within- and between-group changes identified in the selected studies (Table 2).

**Table 2:** Participants' outcome regarding depression status pre- and post-interventions PHQ-9: Patient Health Questionnaire-9; NA: not available; ICBT: internet-based cognitive behavioral therapy; WLC: waitlist control; TAU: treatment as usual; MVC: mindfulness CBT video-based program; iFD: internet-based Functional Depression; PMR: progressive muscle relaxation; ITT: intention-to-treat analysis; M: mean; SD: standard deviation; CI: confidence Interval; SE: standard error of the mean; IDS-SR: Inventory of Depressive Symptomatology-Self-Rating

Study	Country	Pre-intervention			Post-intervention			Comments
		PHQ-9, mean (SD) (treatment)	PHQ-9, mean (SD) (control)	P-value	PHQ-9, mean (SD) (treatment)	PHQ-9, mean (SD) (control)	P-value	
Xiang et al., 2024 [20]	USA	11.49 (2.63)	11.29 (2.65)	p=0.75	5.64 (4.79)	8.84 (4.30)	p<0.001	Empower@Home was more efficacious in reducing depressive symptoms than friendly telephone calls and depression symptom monitoring. Most participants in the intervention group completed all nine sessions (31/35, 89%). In the linear mixed model, the group-by-time interaction was statistically significant (p<0.001), and the usability and acceptability ratings were excellent. The intervention group had a large within-group change in depressive symptoms (Cohen's d=1.22; p<0.001), while the attention control group had only a medium change (Cohen's d=0.57; p<0.001). The between-group effect size was also significantly notable in the intervention over the control group (Cohen's d=0.72; p<0.001). Besides the depressive symptoms, there were enhancements in CBT skills, including self-satisfaction, competence, and behavioral activation. Furthermore, secondary psychosocial outcomes, including anxiety, anger, social isolation, insomnia, and pain, did not experience changes in these outcome domains. However, there were differences owing to the small sample size
Lin et al., 2023 [21]	China	13.10 (5.96)	WLC=12.6 (5.07)	p=0.72	ICBT=6.19 (5.68). Module completers=6.24 (0.82). Non-completers=8.94 (1.87)	WLC=10.88 (5.80)	p<0.001	Compared with the WLC group, the ICBT group had fewer depressive symptoms. There was no statistically significant difference in the PHQ-9 score between completers and non-completers in the ICBT group at the post-treatment assessment
Lopes et al., 2023 [22]	Brazil	19.57 (4.81)	20.12 (4.14)	p=0.46	NA	NA	NA	The study included a severely depressed sample, which is less common in research and strengthens the findings. The results demonstrated higher effect sizes than other studies with severely depressed participants using Deprexis. Participants from the immediate access group logged in at Deprexis an average of 14.81 (SD 12.16) times. Based on the PHQ-9, the ITT analysis (using a linear mixed model) found that Deprexis significantly improved depression and had higher satisfaction levels than the control group (Cohen's d=0.80; p<0.001). Also, the ITT analysis revealed a higher remission rate in the intervention group compared to the control group (p<0.001). Deprexis could effectively improve depressive symptoms in three months for Brazilian users with depression. The study experienced a high dropout rate among randomized participants who did not complete post-treatment questionnaires. Additionally, the intervention group showed a marked development in the perceived self-efficacy measure with a medium effect size
Stuart et al., 2022 [23]	USA	14.6 (95% CI: 13.8-15.5)	14.9 (95% CI: 14.1-15.7)	NA	8.1 (95% CI: 7.0-9.2)	10.9 (95% CI: 9.9-11.9)	p=0.002	ICBT was associated with greater depression response and remission at eight weeks compared with the control group. Depression scores in the intervention group remained similar at 24 weeks, at which time the control group also showed a similar rate of response and remission. The intervention group maintained the eight-week symptom reduction, while the TAU alone group exhibited similar improvements by 24 weeks (ICBT PHQ-9 total score of 8.9 vs TAU score of 9.9). The intervention group saw a relative improvement of 2.5 points in PHQ-9 scores at eight weeks (p=0.002). Thus, the intervention had a statistically significant effect on PHQ-9 total scores (F=6.28, p=0.002), with patients in the ICBT plus TAU group experiencing a moderate mean reduction in PHQ-9 total score at eight weeks (est=-2.54; 95% CI: -4.02, -1.07; d=-0.48) compared to patients in the TAU alone group (ICBT PHQ-9 total score of 14.6 at baseline decreased to 8.1 at eight weeks vs TAU alone score of 14.9 at baseline decreased to 10.9 at eight weeks)
Karyotaki et al., 2022 [24]	Netherlands	8.52 (2.87)	7.96 (2.98)	NA	ITT analysis post-treatment ICBT=7.66 (0.83); six months follow-up=6.63 (0.65); 12 months follow-up=ICBT=6.73 (0.85), 48. Complete cases post-treatment=7.37 (0.78); six months follow-up=6.60 (0.70); 12 months follow-up=6.83 (0.84)	TAU post-treatment=7.68 (0.75); six months follow-up=6.17 (0.59); 12 months follow-up=6.39 (0.68). Complete cases post-treatment=7.78 (0.75); six months follow-up=6.08 (0.54); 12 months follow-up=6.54 (0.64)	Post-treatment: p=0.65; six months follow-up: p=0.74; 12 months follow-up: p=0.87. Complete case: p=0.51; six months follow-up: p=0.75; 12 months follow-up: p=0.90	There was no evidence of a difference between the effects of guided ICBT and TAU in any of the examined outcomes (i.e., symptoms of depression) across all time points (p>0.05)
Lindegaard et al., 2021 [25]	Sweden	15.63 (6.67)	17.79 (5.29)	NA	Week 3=13.81 (5.45). Post-treatment=11.67 (6.05)	Week 3=15.87 (4.76). Post-treatment=17.33 (5.29)	p=0.039	A significant effect was found for group-by-time (-0.42) (95% CI: (-0.82, -0.02); z=-2.06; p=0.039) on the PHQ-9, thus showing that the treatment group improved on average 0.42 points more per week as compared to the control group. In addition, the ITT analysis showed that the treatment group who received ICBT had a significant reduction in depressive symptoms compared to the WLC group. The between-group effect at post-treatment measures was as follows: Cohen's d=0.85 (0.29, 1.41). The results indicate that ICBT is a promising treatment approach for treating symptoms of depression, insomnia, and stress in an Arabic-speaking immigrant population
McCloud et al., 2020 [26]	UK	8.3 (3.73)	8.3 (4.20)	NA	Week 2=6.3 (3.20). Week 4=5.9 (3.63). Week 6=5.8 (3.72)	Week 2=6.9 (3.84). Week 4=6.6 (3.61). Week 6=6.6 (4.07)	Week 2: p=00.16. Week 4: p=0.04. Week 6: p=0.006	At week 6, the primary endpoint, there was evidence that the Feel Stress-Free app reduced depression symptoms (mean difference: -1.56; 95% CI: -2.67 to -0.44; p=0.006), but there was only very weak evidence that it reduced anxiety symptoms (mean difference: -1.36; 95% CI: -2.93 to 0.21; p=0.09) and, though weaker, depression symptoms (mean difference: -1.08; 95% CI: -2.12 to -0.04; p=0.04). At week 6, 83% (34/41) of participants indicated that they were using the app weekly or more frequently. The Feel Stress-Free app is a promising mobile intervention for treating symptoms of anxiety and depression in students and overcomes many of the barriers to traditional CBT. Further research is needed to establish its effectiveness at and beyond six weeks
El Morr et al., 2020 [27]	Canada	8.36 (5.62)	9.91 (6.22)	p=0.10	7.04 (5.03)	11.21 (6.72)	p<0.001	At post-intervention follow-up, according to the adjusted comparisons, there were statistically significant between-group reductions in depression scores (β=-2.21; p=0.01), suggesting that an eight-week-long online MVC is an effective intervention for undergraduate university students in reducing symptoms of depression and anxiety
Oehler et al., 2020 [28]	Germany	9.1 (3.6)	9.7 (3.3)	NA	7.0 (3.7)	6.9 (3.7)	p=0.01	The primary outcome measure was the IDS-SR. The study was rigorously designed with detailed data collection and analysis, including mixed model analyses to handle incomplete data. Over the entire observation period, the iFD group showed a significantly greater reduction in depressive symptoms compared to the PMR group (p=0.01) and a greater improvement in quality of life (p<0.001). Significant effects on depressive symptoms were observed at the three-month follow-up (d=0.281; 95% CI: 0.069-0.493) but not immediately after the six-week intervention (d=0.192; 95% CI: -0.020 to 0.404) or at six and 12 months. The iFD intervention was significantly superior to PMR in terms of user satisfaction (mean score: 25.31 vs 21.97; t=5.804; p<0.01). Separate tests for each time point revealed significant effects on depressive symptoms at the three-month follow-up (d=0.281; 95% CI: 0.069-0.493) but not after six weeks (main outcome: d=0.192; 95% CI: -0.020 to 0.404) and six and 12 months. The use of an active control condition (PMR) rather than a WLC enhances the validity of the findings by reducing potential nocebo effects. The inclusion of a 12-month follow-up period allowed for the assessment of long-term effects, providing valuable insights into the sustained impact of the intervention. The active control condition (PMR) itself may have had antidepressant effects, potentially underestimating the true efficacy of the iFD intervention. The amount of guidance provided was significantly longer in the iFD group compared to the PMR group, which may have influenced the outcomes. The study sample was self-selected, highly educated, and experienced with internet use, which may limit the generalizability of the findings to more diverse populations. The study did not collect data on additional treatments participants may have received during the follow-up period, making it difficult to control for external factors
Johansson et al., 2019 [29]	Sweden	12.3 (4.1)	11.7 (3.7)	NA	6.2 (3.6)	11.1 (2.6)	NA	Measures assessing depression, anxiety, and psychiatric symptoms were administered before and after treatment, and follow-up was performed at six and 12 months after treatment had ended. ICBT resulted in significant reductions in depressive symptoms in the treatment group when compared to a WLC group with a large effect size (Cohen's d=1.6). Treatment gains were maintained six and 12 months after the treatment ended. Regarding clinical significance, 58% of the sample had improved or recovered after treatment. ICBT appears to be an effective treatment for depression when delivered as an integral part of routine psychiatric care. The study was small, and patients received general psychiatric care after the ICBT treatment had ended, which limits the implications
Schure et al., 2019 [30]	USA	13.7 (5.0)	13.4 (5.0)	p<0.001	7.7 (SE 0.336)	10.224 (SE 0.328)	p<0.001	There was a significant reduction in depression symptom severity (PHQ-9 scores) in the Thrive intervention group compared to the control group over the eight weeks (p<0.001). The intervention group had a moderate treatment effect size (Cohen's d=0.63). Moderate to near-moderate effect sizes favoring the intervention group were observed for anxiety symptoms (p<0.001; d=0.47), work functioning, and resilience. The results recommend that the ICBT intervention "Thrive" was successful in lowering the intensity of anxiety and depression symptoms as well as enhancing functioning and resilience in the rural community population of US adults. The study showed that effect sizes for secondary outcomes were higher than in other automated ICBT interventions and included a range of symptoms from mild to severe. These interventions didn't need a clinician, making them useful for underserved areas. However, low follow-up rates meant the study only looked at short-term effects, leaving long-term results unclear. The sample was mostly female and Caucasian, which may limit the findings to more diverse groups. Since all data were self-reported, bias is possible, even with validated tools

ROB Assessment

**Figure 2:**
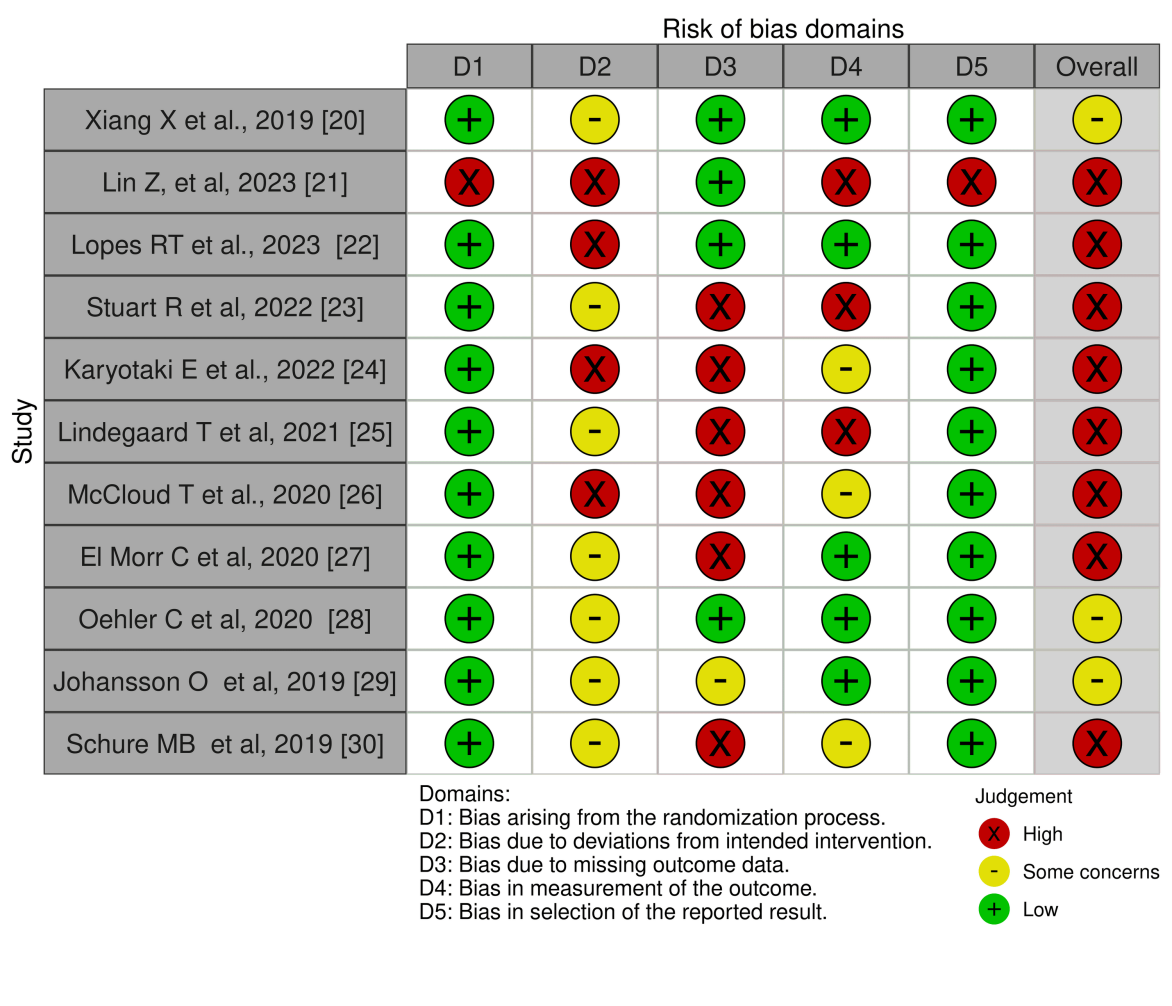
Risk of bias assessment for the included RCT RCT: randomized controlled trial

**Figure 3:**
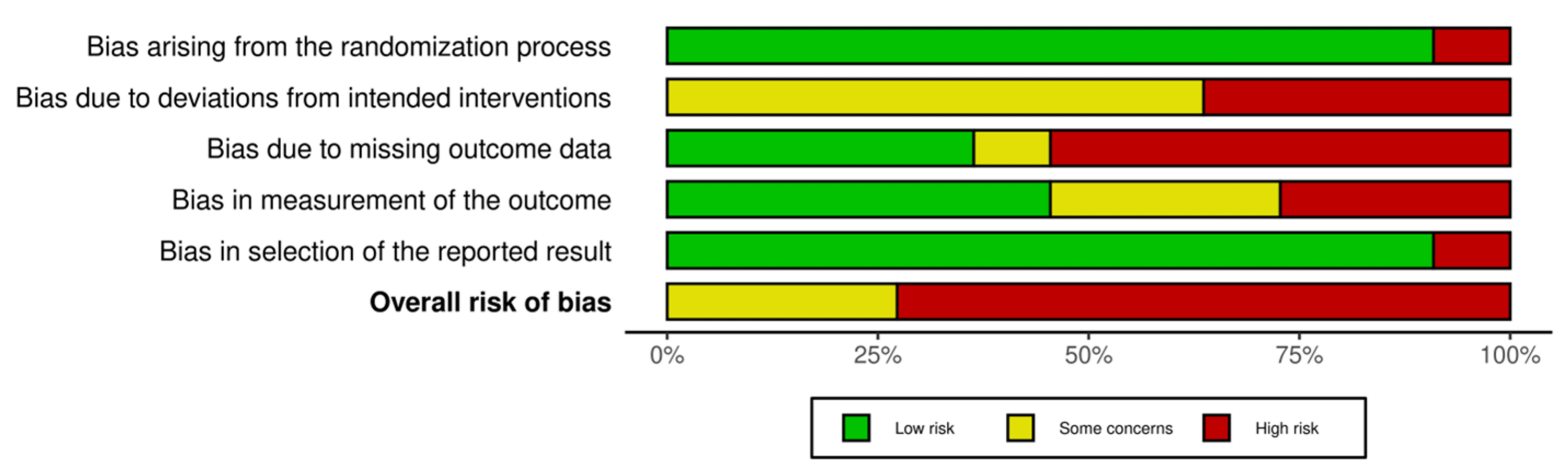
Summary of the risk of bias assessment

Overall, the ROB was high across eight of our included studies. This bias was primarily due to missing outcome data, deviation from the intended intervention, measurement of the outcome, randomization process, and selection of the reported results. In addition, three studies had concerns regarding the deviation from the intended intervention and missing outcome data. We had access to the full databases of the included studies; thus, we could assess all available depression measures. Therefore, 10 trials were at low risk of selective reporting (Figure 2 and Figure 3).

Discussion

This systematic review assessed internet-based interventions, including Empower@Home, Deprexis, guided and self-guided ICBT, the Thrive app, MVC, and iFD. Most studies also used PHQ-9 as an assessment tool to measure treatment efficacy. Only one study used the HADS to evaluate treatment efficacy [26].

Among the included studies, significant improvements were found in most interventions, demonstrating significant reductions in depressive symptoms compared to control groups. This was evident in the following: ICBT (p<0.001, p<0.001, p=0.002, p=0.039) [20,21,23,25,29], Thrive ICBT (p<0.001) [30], Feel Stress-Free app over six and eight weeks (p=0.04, 0.006) [26], mindfulness CBT video-assisted program (p<0.001) [27], and iFD tool (p=0.01) [26]. Xiang et al. found that in the linear mixed model, the group-by-time interaction was statistically significant (p<0.001) when comparing the ICBT program with the control group [20]. The authors revealed that improved CBT skills acquisition, behavioral activation, and satisfaction with the basic psychological needs of autonomy, competence, and relatedness mediated the treatment effects. Lin et al. compared the WLC group with the ICBT group, indicating that fewer depressive symptoms were evident in the intervention group. However, there was no statistically significant difference in the PHQ-9 scores between completers and non-completers in the ICBT group at the post-treatment assessment. This may be because the post-treatment assessment results were lacking, as the authors did not collect and analyze any follow-up data [21]. While Stuart et al. revealed that the integration of the ICBT program was associated with greater depression response and remission by the end of week 8, the TAU group showed similar results by the end of 24 weeks (ICBT PHQ-9 total score of 8.9 vs TAU score of 9.9) [23]. However, the attrition rate was high, and it was difficult to conclude whether the missing participants would have benefited from this intervention. Several studies that implemented ICBT programs have reported varying retention rates [31-36].

The missing follow-up data problem was avoided by McCloud et al. as researchers applied a multilevel modeling analysis technique that allowed all participants to contribute to analyses and found that the Feel Stress-Free program resulted in significant improvement in depression symptoms by week 6 despite the small sample size (p=0.006). Nevertheless, its efficacy over longer periods was not discussed [26]. Interestingly, the mindfulness CBT video-assisted program resulted in a significant reduction in depression symptoms (p<0.001) over eight weeks among undergraduate participants. However, this finding was accompanied by many limitations similar to those of other internet CBT intervention studies. These include the generalizability of the results, blinding issues, and limitations in the application timeline [27].

In addition, Oehler et al. addressed the lack of participant follow-up when examining the iFD program. Researchers found that the iFD program resulted in a greater reduction in symptoms of depression (p=0.01) over three months of follow-up [28]. The different aspect of this study is attributed to its control group, which included active control groups (PMR). This might affect the results, as the active control condition cannot be considered a placebo. Previous research referred to the antidepressant effect of PMR [37] and that possible treatment differences between the iFD and PMR groups during follow-up could not be detected. Lastly, over an eight-week follow-up, the Thrive ICBT intervention resulted in significantly lower depression symptoms (p<0.001), and having a fully automated nature empowered the effect size [30]. However, as with previous ICBT programs, problems in follow-up, missing outcomes, and generalizability could not be avoided.

Despite the significant effect of the ICBT program demonstrated in Johansson et al.'s study, similar limitations regarding this program were also addressed [29]. The ICBT program should be adjusted for each population to avoid different types of biases. It is recommended to employ this intervention among larger sample sizes and longer periods with follow-up to estimate its true efficacy among depressed individuals. It is worth noting that this program has proved its efficacy among vulnerable populations among a pilot RCT, meaning that more studies should be conducted among larger variable populations [25].

In contrast to the current findings, only one study of ours showed no evidence of a difference between the effects of guided ICBT and TAU in any of the examined outcomes of depression across all time points [24]. This could be because many reasons influence the significance of the findings, including inflated outcomes due to control conditions, differences in baseline symptom severity, intervention format, and guidance.

Strengths

Our review provides valuable insights into the literature, as this review included studies with a wide range of proportions of using antidepressants, which amplifies the findings. Also, the studies used standardized scales for depression, which offer a valid comparison of outcomes and provide strong evidence for the efficacy of web-based CBT interventions.

Limitations

According to our findings in this systematic review, there are several limitations exist among the included studies that could affect our findings from reaching conclusive results. For example, biases, generalizability issues, low sample size, missing outcome data, and loss of follow-up were among the most common issues in the studies. For instance, CBT for acute treatment usually lasts 12-16 weeks [38]. However, the intervention of ICBT programs for 6-8 weeks was mostly used in the included studies, which were considered less effective and requiring longer duration.

Future Directions

Longitudinal studies with extended follow-up periods are required to assess the effect of ICBT programs on the risk of depression relapse. Additionally, future studies related to mental health issues should involve digital intervention for the best practice and clinical outcomes for depressive patients.

## Conclusions

This systematic review aimed to estimate the efficacy of several types of web-based interventions among patients with depression symptoms using quantifiable and reliable assessments. Despite the significance of most interventions favoring internet-based interventions compared to control groups, many limitations existed in the included studies, making it unclear whether the interventions would prove conclusive benefits. Therefore, future studies should aim to enhance the validity of the findings. For instance, it is significant to investigate the mid- and long-term effects of ICBT. Researchers could consider adapting the content of ICBT interventions and extending their duration to achieve sustained and more pronounced relief of depressive symptoms while ensuring participant compliance. In addition, collecting more detailed data on participants' usage of the online applications, including qualitative feedback on their perceived usefulness and user experience, would be beneficial. Direct monitoring of participants' app usage is crucial to determine if there is a dose-response relationship and whether certain activities within the app are associated with better outcomes.
